# Quantum Security of Nonce-Based Encryption

**DOI:** 10.3390/e27121194

**Published:** 2025-11-24

**Authors:** Shuping Mao, Peng Wang, Yan Jia, Gang Liu, Bing Liu

**Affiliations:** 1 Beijing Electronic Science & Technology Institute, Beijing 100070, China; maoshuping19@mails.ucas.ac.cn (S.M.); leoliu76@outlook.com (B.L.); 2 School of Cryptology, University of Chinese Academy of Sciences, Beijing 100049, China; p-wang@ucas.ac.cn; 3 State Key Laboratory of Cyberspace Security Defense, Institute of Information Engineering, Chinese Academy of Sciences, Beijing 100085, China; jiayan22@mails.ucas.ac.cn; 4 School of Cyber Security, University of Chinese Academy of Sciences, Beijing 100049, China; 5 National Key Laboratory of Security Communication, Chengdu 610041, China

**Keywords:** nonce-based encryption, R-IND-qCPA, N-IND-qCPA

## Abstract

We investigate the quantum security of nonce-based encryption under the indistinguishability against quantum chosen-plaintext attacks (IND-qCPA). While classical results establish that IV-based modes such as CBC, CFB, OFB, and CTR achieve IND-qCPA security, we demonstrate that simply replacing the random IV with a nonce undermines both classical and quantum security. To address this, we propose a general transformation from R-IND-qCPA security to N-IND-qCPA security and introduce enhanced variants, namely, CBC2, CFB2, OFB2, and CTR2, that are provably secure in the nonce-based quantum setting. We further show that nonce-based stream cipher encryption inherently satisfies N-IND-qCPA security. These results provide a systematic framework for upgrading IV-based constructions to secure nonce-based counterparts, thereby strengthening practical symmetric encryption against quantum adversaries.

## 1. Introduction

A block cipher is a basic building block in modern cryptography, usually modeled as a random permutation on fixed-length inputs. In practice, messages are often much longer than a single block and may vary in length, which requires the use of block cipher modes of operation to extend encryption to arbitrary-length inputs. Modes of operation are generally divided into three categories: encryption modes, authentication modes, and authenticated encryption modes. The earliest encryption modes, such as ECB, CBC, OFB, and CFB, provided confidentiality only. To ensure integrity, authentication modes such as CBC-MAC were developed, and later integrated designs such as CCM, GCM, and OCB were proposed to achieve both confidentiality and integrity.

The progress of quantum algorithms has posed serious challenges to these modes. When adversaries are allowed quantum query access to the underlying primitive, algorithms such as Grover’s search [1] and Simon’s algorithm [2] can be applied to mount attacks. Under this setting, reduced-round Feistel and Lai–Massey structures, the Even–Mansour cipher, LRW [3,4,5,6,7], authentication modes including CBC-MAC, PMAC, and GMAC [7], and authenticated encryption modes such as OCB and GCM [7,8,9,10] have been shown to be insecure.

Against this background, the quantum security of encryption modes has become a central research topic. The natural adaptation of the classical indistinguishability under chosen-plaintext attack (IND-CPA) model to the quantum setting is the IND-qCPA model [11], where challenge queries remain classical, encryption queries may be quantum, and the adversary’s distinguishing advantage quantifies security. Because encryption modes aim only at confidentiality, proving IND-qCPA security suffices in the quantum setting. It is important to note that existing quantum security models are probabilistic, in which a random value *r* is included during the encryption process. Unlike plaintexts and ciphertexts, which may be in quantum superposition, the random value *r* is always treated as a classical number.

Based on the nature of the random value *r*, encryption schemes can be categorized into IV-based encryption schemes and nonce-based encryption schemes. Among these, an initialization vector IV is required to be random, whereas a nonce *N* is required only to be arbitrarily chosen but non-repeating. Compared with random IVs, nonces are easier to generate, avoid reliance on high-quality randomness, and better match practical deployment requirements in modern cryptographic protocols.

The quantum security of encryption schemes varies depending on the type of random value employed. In 2016, Vivekanand et al. [12] demonstrated that encryption modes such as CBC, CFB, OFB, and CTR achieve IND-qCPA security when a random IV is used. In 2021, Bhaumik et al. [8] proposed a new design called QCB, based on OCB, and proved that QCB satisfies IND-qCPA security under the non-adaptive nonce setting. Here, a non-adaptive nonce refers to one that may be arbitrarily chosen but must be selected entirely in advance prior to any encryption queries. In 2025, Lang et al. [13] provided the first formal definition of IND-qCPA security in the adaptive nonce model, although no concrete instantiation has been proposed to date. In addition, there are also studies on the quantum security of block ciphers and their modes of operation [14,15,16,17].

We refer to IV-based IND-qCPA as R-IND-qCPA and nonce-based IND-qCPA as N-IND-qCPA. Although CBC, CFB, OFB, and CTR achieve IND-qCPA security when a random IV is used (that is, they are R-IND-qCPA secure), Section 3 demonstrates that replacing the IV with a nonce *N* not only fails to provide IND-qCPA security but also undermines even IND-CPA security. Enhancing encryption schemes to achieve N-IND-qCPA security constitutes a primary focus of this work.

The main results in the paper are as follows (Table 1):

1. We present a general transformation that converts any R-IND-qCPA secure encryption scheme into an N-IND-qCPA secure scheme.
2. We propose enhanced variants of CBC, CFB, OFB, and CTR, referred to as CBC2, CFB2, OFB2, and CTR2, and provide formal proofs of their security under the N-IND-qCPA definition.
3. We further show that nonce-based stream cipher encryption inherently satisfies N-IND-qCPA security.

The structure of this paper is organized as follows: Section 2 introduces definitions and notation; Section 3 presents N-IND-CPA attacks against CBC, CFB, OFB, and CTR; Section 4 provides the N-IND-qCPA security proof for CBC2; Section 5 discusses improvements and proofs for other encryption schemes; and Section 6 offers concluding remarks.

## 2. Preliminaries

### 2.1. Notations

**Block Cipher.** A block cipher $E:{\left\{0,1\right\}}^{k}\times {\left\{0,1\right\}}^{n}\to {\left\{0,1\right\}}^{n}$ is a map with key space ${\left\{0,1\right\}}^{k}$ and message space ${\left\{0,1\right\}}^{n}$ such that for every key $K\in {\left\{0,1\right\}}^{k}$, P↦E(K,P) is a permutation on ${\left\{0,1\right\}}^{n}$. Let $E_{K}$ denote the map P↦E(K,P).

Let $x\overset{\$}{\leftarrow }{\left\{0,1\right\}}^{n}$ denote selecting an element *x* from the set ${\left\{0,1\right\}}^{n}$ uniformly at random. Let Perm(n) be a set of all permutations on ${\left\{0,1\right\}}^{n}$. Let $\pi \overset{\$}{\leftarrow }\mathrm{Perm}\left(n\right)$ be a random permutation on ${\left\{0,1\right\}}^{n}$. A block cipher keyed by *K* is a function $E_{K}\in \mathrm{Perm}\left(n\right)$. We call the input and output of $E_{K}$ as plaintext and ciphertext, respectively. Let Func(m,n) be the set of all functions from ${\left\{0,1\right\}}^{m}$ to ${\left\{0,1\right\}}^{n}$. We write Func(n,n) as Func(n).

**IV-Based Encryption Scheme.** Encryption schemes are typically defined as either probabilistic or stateful. In the context of symmetric cryptography, the randomness or state involved in the encryption process is usually represented explicitly by an initialization vector (IV), meaning that both encryption and decryption can be formalized as deterministic algorithms. The following provides the syntax definition of an IV-based encryption scheme. An IV-based encryption scheme is a pair of algorithms Π=(Enc,Dec), where Enc:Key×IV×Plaintext→Ciphertext and Dec:Key×IV×Ciphertext→Plaintext are deterministic functions with key space, IV space IV, plaintext space Plaintext, and ciphertext space Ciphertext. We require that $Dec(K,IV,Enc_{K}\left(IV,P\right))=P$ for any K∈Key and IV∈IV and P∈Plaintext. Note that here, for a probabilistic encryption scheme IV is randomly selected from IV.
**Nonce-Based Encryption Scheme.** If the only requirement on the initialization vector (IV) is that it must not repeat, then even allowing the adversary to choose the IV is acceptable. In this case, the IV is referred to as a nonce, and the corresponding construction is called a nonce-based encryption scheme. Because the syntax of IV-based and nonce-based encryption schemes is identical, the latter simply relaxes the requirements on how the IV is generated. This relaxation greatly facilitates the secure deployment of symmetric encryption, as producing a random IV would otherwise require the additional implementation of a secure random number generator. To highlight the distinction, in the syntax definition the IV space IV is usually replaced by a nonce space Nonce.

### 2.2. Security Definitions

Let A be an adversary. Let $A^{O}=b$ denote an algorithm that performs queries on the oracle O and produces the bit of *b*. In the context of a (keyed) function *f*, for a classical query *X*, the response is the value f(X). In contrast, a quantum query is given as a quantum superposition state $\sum \psi_{X,Y}|X\rangle |Y\rangle$, and the response is given by $\sum \psi_{X,Y}|X\rangle |Y⊕f\left(X\right)\rangle$.

For two oracles $O_{1}$ and $O_{2}$, the classical and quantum distinguishing advantage of A is defined respectively as follows:

$$\begin{array}{cc} \; & {\mathrm{Adv}}_{O_{1},O_{2}}^{\mathrm{dist}}\left(A\right):=\left|\mathrm{Pr}\left[A^{O_{1}\left(\cdot \right)}=1\right]-\mathrm{Pr}\left[A^{O_{2}\left(\cdot \right)}=1\right]\right|,\\ \; & {\mathrm{Adv}}_{O_{1},O_{2}}^{\mathrm{qdist}}\left(A\right):=\left|\mathrm{Pr}\left[A^{O_{1}\left(*\right)}=1\right]-\mathrm{Pr}\left[A^{O_{2}\left(*\right)}=1\right]\right|, \end{array}$$

where we use · to denote a classical query and ⊙ to denote a quantum query.

**Definition 1**  (PRF/qPRF). *A (quantum-secure) pseudorandom function ((q)PRF) is an efficiently computable function family $f:{\left\{0,1\right\}}^{k}\times {\left\{0,1\right\}}^{s}\to {\left\{0,1\right\}}^{n}$ for all (quantum) algorithms A,*

$$\begin{array}{c} \left|\underset{k\overset{\$}{\leftarrow }{\left\{0,1\right\}}^{k}}{\mathrm{Pr}}\left[A^{f_{k}}\left(*\right)=1\right]-\underset{g\overset{\$}{\leftarrow }\mathrm{Func}\left(s,n\right)}{\mathrm{Pr}}\left[A^{g}\left(*\right)=1\right]\right|\leq \mathrm{negl}, \end{array}$$

*where g is a random function from ${\left\{0,1\right\}}^{s}$ to ${\left\{0,1\right\}}^{n}$ and where we replace the ∗ symbol by · to denote a classical query or *⊙* to denote a quantum query).*

**Definition 2**  (PRF/qPRF/PRP/qPRP Advantages). *Let $F:{\left\{0,1\right\}}^{k}\times {\left\{0,1\right\}}^{s}\to {\left\{0,1\right\}}^{n}$ be a function. Let $E:{\left\{0,1\right\}}^{k}\times {\left\{0,1\right\}}^{n}\to {\left\{0,1\right\}}^{n}$ be a block cipher. Let f←Func(s,n) be a random function. Let p←Perm(n) be a random permutation. We assume that all keys are random. The PRF/qPRF/PRP/qPRP advantages are defined as follows:*

$$\begin{array}{c} {\mathrm{Adv}}_{F}^{\mathrm{PRF}\;}\left(A\right)={\mathrm{Adv}}_{F_{K},f}^{\mathrm{dist}}\left(A\right),\\ {\mathrm{Adv}}_{F}^{\mathrm{qPRF}\;}\left(A\right)={\mathrm{Adv}}_{F_{K},f}^{\mathrm{qdist}}\left(A\right),\\ {\mathrm{Adv}}_{E}^{\mathrm{PRP}\;}\left(A\right)={\mathrm{Adv}}_{E_{K},\pi }^{\mathrm{dist}}\left(A\right),\\ {\mathrm{Adv}}_{E}^{\mathrm{qPRP}}\left(A\right)={\mathrm{Adv}}_{E_{K},\pi }^{\mathrm{qdist}}\left(A\right). \end{array}$$

**Definition 3**  (Secure Stream Cipher). *A secure stream cipher is an efficiently computable function $SC:{\left\{0,1\right\}}^{k}\times {\left\{0,1\right\}}^{s}\to {\left\{0,1\right\}}^{L}$ that takes a key k and nonce N, then outputs a keystream of length L. For all probabilistic polynomial-time adversaries A, the following advantage is negligible:*

$$\begin{array}{c} \left|\underset{K\overset{\$}{\leftarrow }{\left\{0,1\right\}}^{k}}{\mathrm{Pr}}\left[A^{SC_{K}\left(\cdot \right)}=1\right]-\underset{g\overset{\$}{\leftarrow }\mathrm{Func}\left(s,L\right)}{\mathrm{Pr}}\left[A^{g}\left(\cdot \right)=1\right]\right|\leq \mathrm{negl}, \end{array}$$

*where g is a random function from ${\left\{0,1\right\}}^{s}$ to ${\left\{0,1\right\}}^{L}$.*

In the following, we present the definitions of R-IND-CPA and N-IND-CPA.

**Definition 4**  (R-IND-CPA). *For an encryption scheme Π=(Enc,Dec) and an adversary A, we define the advantage of indistinguishability under a chosen plaintext attack in the random-IV setting (R-IND-CPA) using the following game:*

***Key Generation**: The challenger picks a random key K and a random bit b.*
***Queries**: A is allowed to make two types of queries:*
  **Challenge Queries**: A sends two plaintexts $P_{0},P_{1},$ to which the challenger chooses randomness R and responds with $C^{*}=R∥{\mathrm{Enc}}_{K}\left(R,P_{b}\right)$.
  ***Encryption Queries**: For each such query of P, the challenger chooses randomness R and responds with $C=R∥{Enc}_{K}\left(R,P\right)$.*
***Guess**: A produces a bit $b^{\prime }$ and wins if $b=b^{\prime }$.*

*The R-IND-CPA advantage of an adversary A is defined as*

$$\begin{array}{c} {\mathrm{Adv}}_{\Pi }^{R-\mathrm{IND}-\mathrm{CPA}}\left(A\right)=\left|2\mathrm{Pr}[A\;\mathrm{success}\;]-1\right|. \end{array}$$

**Definition 5**  (N-IND-CPA). *For an encryption scheme Π=(Enc,Dec) and an adversary A, we define the advantage of indistinguishability under a chosen plaintext attack in the nonce setting (N-IND-CPA) using the following game:*

***Key Generation**: The challenger picks a random key K and a random bit b.*
***Queries**: A is allowed to make two types of queries:*
  **Challenge Queries**: A sends a nonce N and two plaintexts $P_{0},P_{1}$, to which the challenger responds with $C^{*}={\mathrm{Enc}}_{K}\left(N,P_{b}\right)$.
  ***Encryption Queries**: For each such query of (N,P), the challenger chooses randomness R and responds with $C=R∥{Enc}_{K}\left(N,P\right)$.*
***Guess**: A produces a bit $b^{\prime }$, and wins if $b=b^{\prime }$.*

*We stress that the nonce N in the above game never repeats. The N-IND-CPA advantage of an adversary A is defined as*

$$\begin{array}{c} {\mathrm{Adv}}_{\Pi }^{N-\mathrm{IND}-\mathrm{CPA}}\left(A\right)=\left|2\mathrm{Pr}[A\;\mathrm{success}\;]-1\right|. \end{array}$$

In the following, we define R-IND-qCPA and N-IND-qCPA.

**Definition 6**  (R-IND-qCPA). *For an encryption scheme Π=(Enc,Dec) and an adversary A, we define the advantage of indistinguishability under a quantum chosen plaintext attack in the random-IV setting (R-IND-qCPA) using the following game:*

***Key Generation**: The challenger picks a random key K and a random bit b.*
***Queries**: A is allowed to make two types of queries:*
  **Challenge Queries**: A sends two classical messages $P_{0},P_{1}$, to which the challenger chooses classical randomness R and responds with classical $C^{*}=R∥{\mathrm{Enc}}_{K}\left(R,P_{b}\right)$.
  ***Encryption Queries**: For each such query of P, the challenger chooses classical randomness R and encrypts each plaintext in the quantum superposition using R as the randomness:*
    $$\begin{array}{c} \sum_{P,C}\psi_{P,C}|P,C\rangle \to \sum_{P,C}\psi_{P,C}|P,C⊕\left(R∥{\mathrm{Enc}}_{K}\left(R,P\right)\right)\rangle . \end{array}$$
***Guess**: A produces a bit $b^{\prime }$ and wins if $b=b^{\prime }$.*

*The R-IND-qCPA advantage of an adversary A is defined as*

$$\begin{array}{c} {\mathrm{Adv}}_{\Pi }^{R-\mathrm{IND}-\mathrm{qCPA}}\left(A\right)=\left|2\mathrm{Pr}[A\;\mathrm{success}\;]-1\right|. \end{array}$$

**Definition 7**  (N-IND-qCPA). *For an encryption scheme Π=(Enc,Dec) and an adversary A, we define the advantage of indistinguishability under a quantum chosen plaintext attack in the nonce setting (N-IND-qCPA) using the following game:*

***Key Generation**: The challenger picks a random key K and a random bit b.*
***Queries**: A is allowed to make two types of queries:*
  **Challenge Queries**: A sends a classical nonce N and two classical messages $P_{0},P_{1}$, to which the challenger responds with classical $C^{*}={\mathrm{Enc}}_{K}\left(N,P_{b}\right)$.
  ***Encryption Queries**: For each such query of a classical nonce N and a plaintext in the quantum superposition, the challenger encrypts using the following transformation:*
    $$\begin{array}{c} \sum_{P,C}\psi_{P,C}|P,C\rangle \to \sum_{P,C}\psi_{P,C}|P,C⊕{\mathrm{Enc}}_{K}\left(N,P\right)\rangle . \end{array}$$
***Guess**: A produces a bit $b^{\prime }$ and wins if $b=b^{\prime }$.*

*The N-IND-qCPA advantage of an adversary A is defined as*

$$\begin{array}{c} {\mathrm{Adv}}_{\Pi }^{N-\mathrm{IND}-\mathrm{qCPA}}\left(A\right)=\left|2\mathrm{Pr}[A\;\mathrm{success}\;]-1\right|. \end{array}$$

### 2.3. Encryption Modes

**Figure 1:**
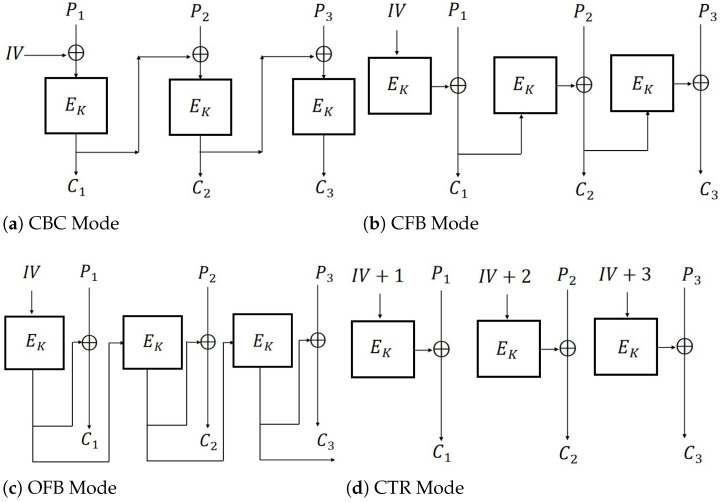
Encryption modes (m=3).

The encryption schemes CBC, CFB, OFB, and CTR (Figure 1) are defined as follows:

**Definition 8**  (CBC Mode). *Let K∈Key and IV∈IV. For a given message $P=P_{1}P_{2}\ldots P_{m}$, where $P_{i}\left(i=1,2,\ldots ,m\right)$ is a block of the message, the symmetric encryption scheme CBC is defined as follows:*

*Enc: $C_{0}=IV$ and $C_{i}=E_{K}\left(P_{i}⊕C_{i-1}\right)$ for 1≤i≤m. En$c_{K}\left(P\right)=C_{0}∥C_{1}\ldots C_{m}$.*
*Dec: For a given ciphertext $C=C_{1}\ldots C_{m}$ and $C_{0}=IV$, $P_{i}:=E_{K}^{-1}\left(C_{i}\right)⊕C_{i-1}$ for 1≤i≤m. De$c_{K}\left(C\right)=P_{1}\ldots P_{m}$.*

**Definition 9**  (CFB Mode). *Let K∈Key and IV∈IV. For a given message $P=P_{1}P_{2}\ldots P_{m}$, where $P_{i}\left(i=1,2,\ldots ,m\right)$ is a block of the message, the symmetric encryption scheme CFB is defined as follows:*

*Enc: $C_{0}=IV$ and $C_{i}=E_{K}\left(C_{i-1}\right)⊕P_{i}$ for 1≤i≤m. En$c_{K}\left(P\right)=C_{0}∥C_{1}\ldots C_{m}$.*
*Dec: For a given ciphertext $C=C_{1}\ldots C_{m}$ and $C_{0}=IV$, $P_{i}:=E_{K}\left(C_{i-1}\right)⊕C_{i}$ for 1≤i≤m. De$c_{K}\left(C\right)=P_{1}\ldots P_{m}$.*

**Definition 10**  (OFB Mode). *Let K∈Key and IV∈IV. For a given message $P=P_{1}P_{2}\ldots P_{m}$, where $P_{i}\left(i=1,2,\ldots ,m\right)$ is a block of the message, the symmetric encryption scheme OFB is defined as follows:*

*Enc: $C_{0}=R_{0}=IV$, $R_{i}=E_{K}\left(R_{i-1}\right)$ and $C_{i}=R_{i-1}⊕P_{i}$ for 1≤i≤m. En$c_{K}\left(P\right)=C_{0}∥C_{1}\ldots C_{m}$.*
*Dec: For a given ciphertext $C=C_{1}\ldots C_{m}$ and $C_{0}=IV$, $P_{i}:=E_{K}\left(C_{i-1}\right)⊕C_{i}$ for 1≤i≤m. De$c_{K}\left(C\right)=P_{1}\ldots P_{m}$.*

**Definition 11**  (CTR Mode). *Let K∈Key and IV∈IV. For a given message $P=P_{1}P_{2}\ldots P_{m}$, where $P_{i}\left(i=1,2,\ldots ,m\right)$ is a block of the message, the symmetric encryption scheme CTR is defined as follows:*

*1.*  *Enc: $C_{0}=IV$ and $C_{i}=E_{K}\left(C_{0}+i\right)⊕P_{i}$ for 1≤i≤m. En$c_{K}\left(P\right)=C_{0}∥C_{1}\ldots C_{m}$.*
*2.*  *Dec: For a given ciphertext $C=C_{1}\ldots C_{m}$ and $C_{0}=IV$, $P_{i}:=E_{K}\left(C_{0}+i\right)⊕C_{i}$ for 1≤i≤m. De$c_{K}\left(C\right)=P_{1}\ldots P_{m}$.*

According to [12], CBC, CFB, OFB and CTR are R-IND-qCPA secure, and therefore R-IND-CPA secure.

**Theorem 1**  (Theorem 3 and Theorem 4 in [12]). *If the function E is a quantum secure PRF, then CBC, CFB, OFB, and CTR are R-IND-qCPA secure.*

However, our research will indicate that none of them are N-IND-CPA secure, and therefore not N-IND-qCPA secure.

## 3. N-IND-CPA/N-IND-qCPA Attacks with Nonce-Based Encryption Scheme

We noticed that CBC, CFB, OFB, and CTR only maintain the security of Theorem 1 when $C_{0}$ is a random IV. If we replace IV with nonce *N* (which can be selected but not repeated), then CBC, CFB, OFB, and CTR do not even maintain classical security; therefore, they are not N-IND-qCPA secure. For convenience, we define $P_{i}^{j}$ as the *i*-th block in the *j*-th query. It is easy to argue that the advantage of these attacks is 1.

**Figure 2:**
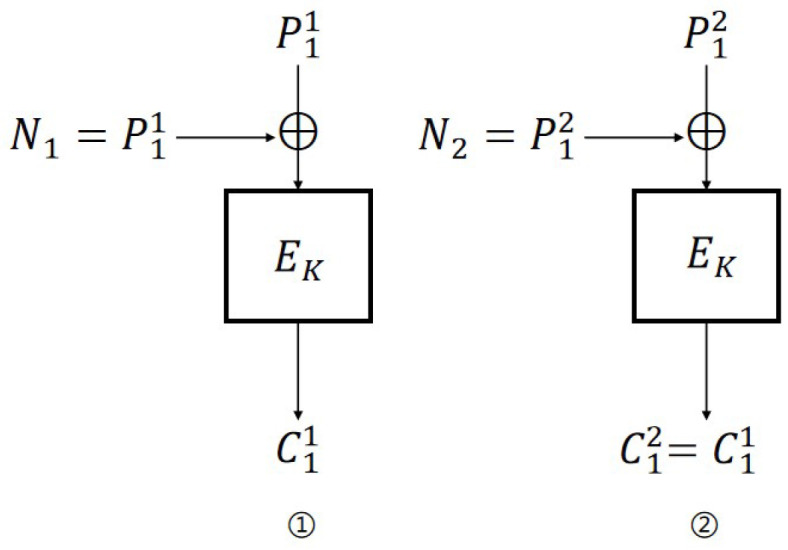
IND-CPA attack on CBC scheme.

**IND-CPA attack on CBC mode.** The IND-CPA attack on CBC is similar to the attack in [18]. The specific process (Figure 2) is as follows:

1. Encryption query: Let $N_{1}=P_{1}^{1}$; then, $C_{1}^{1}=E_{K}\left(0^{n}\right)$.
2. Challenge query: Let $P_{0}=P_{1}^{2}\neq P_{1}^{1}$, $P_{1}\overset{\$}{\leftarrow }{\left\{0,1\right\}}^{n}$, $P_{1}\neq P_{0}$, and $N_{2}=P_{1}^{2}$; then, $b^{\prime }=0$ if $C_{1}^{2}=C_{1}^{1}$, otherwise $b^{\prime }=1$.

For CBC mode, as long as $N_{2}=P_{1}^{2}$, there will be $C_{1}^{2}=C_{1}^{1}$; thus, the probability of adversary A’s success is 1. The distinguishing advantages are: ${\mathrm{Adv}}_{CBC}^{N-\mathrm{IND}-\mathrm{CPA}}\left(A\right)=1$. Therefore, ${\mathrm{Adv}}_{CBC}^{N-\mathrm{IND}-\mathrm{qCPA}}\left(A\right)=1$.

**Figure 3:**
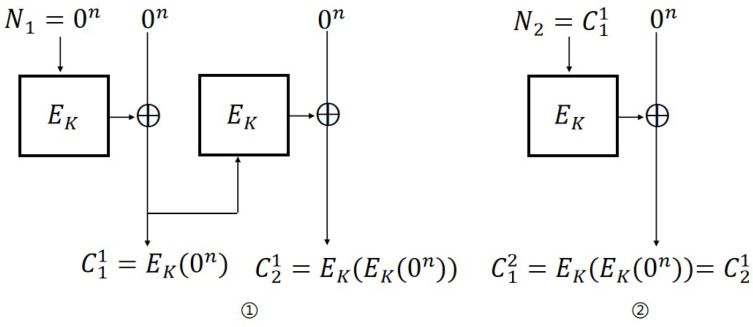
IND-CPA attack on CFB scheme.

**IND-CPA attack on CFB mode.** The steps of the IND-CPA attack (Figure 3) against the CFB scheme are as follows:

1. Encryption query: Let $N_{1}=P_{1}^{1}=P_{2}^{1}=0$; then, $C_{1}^{1}=E_{K}\left(0^{n}\right)$ and $C_{2}^{1}=E_{K}\left(E_{K}\left(0^{n}\right)\right)$.
2. Challenge query: Let $P_{0}=0^{n}$, $P_{1}\overset{\$}{\leftarrow }{\left\{0,1\right\}}^{n}$, $P_{1}\neq P_{0}$, and $N_{2}=C_{1}^{1}=E_{K}\left(0^{n}\right)$; then, $b^{\prime }=0$ if $C_{1}^{2}=C_{1}^{1}$, otherwise $b^{\prime }=1$.

For CFB mode, the probability of adversary A’s success is 1. The distinguishing advantages are: ${\mathrm{Adv}}_{CFB}^{N-\mathrm{IND}-\mathrm{CPA}}\left(A\right)=1$. Therefore, ${\mathrm{Adv}}_{CFB}^{N-\mathrm{IND}-\mathrm{qCPA}}\left(A\right)=1$.

**Figure 4:**
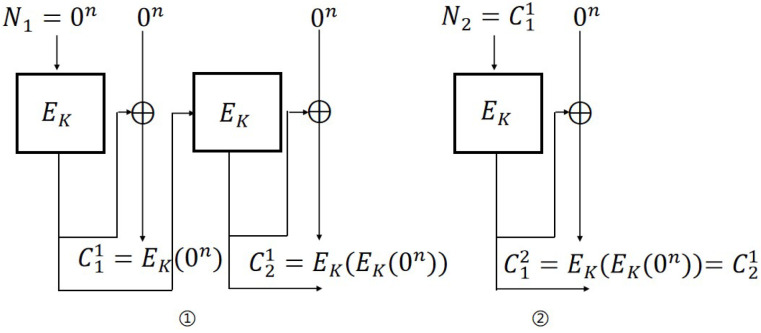
IND-CPA attack on OFB scheme.

**IND-CPA attack on OFB mode.** The steps of the IND-CPA attack (Figure 4) against the OFB scheme are as follows:

1. Encryption query: Let $N_{1}=P_{1}^{1}=P_{2}^{1}=0$; then, $C_{1}^{1}=E_{K}\left(0^{n}\right)$ and $C_{2}^{1}=E_{K}\left(E_{K}\left(0^{n}\right)\right)$.
2. Challenge query: Let $P_{0}=0^{n}$, $P_{1}\overset{\$}{\leftarrow }{\left\{0,1\right\}}^{n}$, $P_{1}\neq P_{0}$ and $N_{2}=C_{1}^{1}=E_{K}\left(0^{n}\right)$; then, $b^{\prime }=0$ if $C_{1}^{2}=C_{1}^{1}$, otherwise $b^{\prime }=1$.

For OFB mode, the probability of adversary A’s success is 1. The distinguishing advantages are: ${\mathrm{Adv}}_{OFB}^{N-\mathrm{IND}-\mathrm{CPA}}\left(A\right)=1$. Therefore, ${\mathrm{Adv}}_{OFB}^{N-\mathrm{IND}-\mathrm{qCPA}}\left(A\right)=1$.

**Figure 5:**
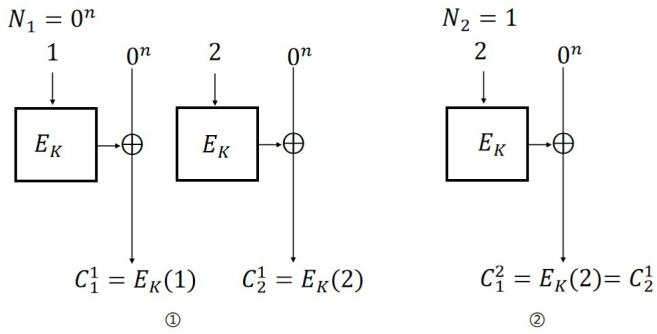
IND-CPA attack on CTR scheme.

**IND-CPA attack on CTR mode.** The steps of the IND-CPA attack (Figure 5) against the CTR scheme are as follows:

1. Encryption query: Let $N_{1}=P_{1}^{1}=P_{2}^{1}=0$; then, $C_{1}^{1}=E_{K}\left(1\right)$ and $C_{2}^{1}=E_{K}\left(2\right)$.
2. Challenge query: Let $P_{0}=0^{n}$, $P_{1}\overset{\$}{\leftarrow }{\left\{0,1\right\}}^{n}$, $P_{1}\neq P_{0}$, and $N_{2}=1$; then, $b^{\prime }=0$ if $C_{1}^{2}=C_{1}^{1}$, otherwise $b^{\prime }=1$.

For CTR mode, the probability of adversary A’s success is 1. The distinguishing advantages are: ${\mathrm{Adv}}_{CTR}^{N-\mathrm{IND}-\mathrm{CPA}}\left(A\right)=1$. Therefore, ${\mathrm{Adv}}_{CTR}^{N-\mathrm{IND}-\mathrm{qCPA}}\left(A\right)=1$.

## 4. CBC2 Mode Is N-IND-qCPA Secure

**Figure 6:**
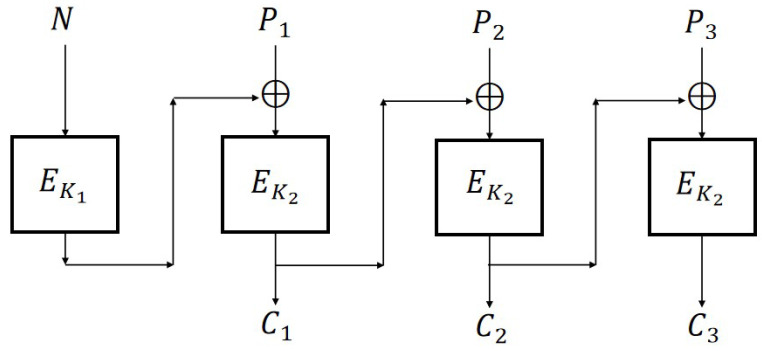
CBC2 scheme (m=3).

From Section 3, we know that CBC, CFB, OFB, and CTR are IND-qCPA secure when based on IV, while they are classical insecure when based on nonce. Based on CBC, Rogaway [18] proposed an improved version of CBC2 (Figure 6) which can achieve N-IND-CPA security by adding a key.

**Definition 12**  (CBC2 Scheme [18]). *Let $K_{1},K_{2}\overset{\$}{\leftarrow }{\left\{0,1\right\}}^{k}$. For a given message $P=P_{1}P_{2}\ldots P_{m}$ and nonce N, where m is a polynomial in n, the symmetric encryption scheme CBC2 is defined as follows:*

*Enc: $C_{0}=E_{K_{1}}\left(N\right)$ and $C_{i}=E_{K_{2}}\left(P_{i}⊕C_{i-1}\right)$ for 1≤i≤m. En$c_{K_{1},K_{2}}\left(P\right)=C_{1}\ldots C_{m}$.*
*Dec: For a given ciphertext $C=C_{1}\ldots C_{m}$ and N, $C_{0}=E_{K_{1}}\left(N\right)$, $P_{i}:=E_{K_{2}}^{-1}\left(C_{i}\right)⊕C_{i-1}$ for 1≤i≤m. De$c_{K_{1},K_{2}}\left(C\right)=P_{1}\ldots P_{m}$.*

For CBC2 scheme, the following theorem holds:

**Theorem 2**  (Theorem 1 in [18]). *If the function E is a secure PRP, then CBC2 is N-IND-CPA secure.*

Next, we will demonstrate that CBC2 also satisfies N-IND-qCPA security in quantum environments:

**Theorem 3.**  *If the function E is a quantum secure PRF, then CBC2 is N-IND-qCPA secure.*

**Figure 7:**
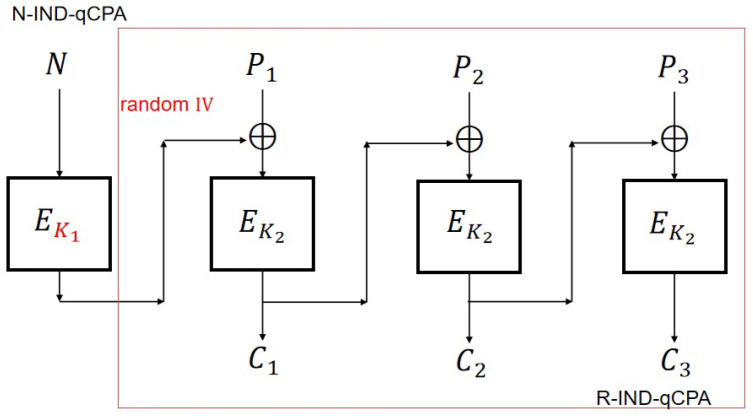
CBC2 scheme and CBC scheme.

From Figure 7, it can be seen that in CBC2, the nonce *N* is first encrypted using $E_{K_{1}}$ and the output at this time is a random value, which can be essentially understood as transforming the adaptive nonce into a random IV through one encryption.

The following theorem ensures the validity of Theorem 3:

**Theorem 4**  (From R-IND-qCPA secure to N-IND-qCPA secure.). *If the function E is a quantum secure PRF, $K_{1},K_{2}\overset{\$}{\leftarrow }{\left\{0,1\right\}}^{k}$, then encryption scheme $Enc_{K_{2}}\left(IV,P\right)$ is R-IND-qCPA secure with classical random IV. Let $Enc_{K_{1},K_{2}}^{\prime }\left(N,P\right)=Enc_{K_{2}}\left(E_{K_{1}}\left(N\right),P\right)$; then, $Enc_{K_{1},K_{2}}^{\prime }\left(N,P\right)$ is N-IND-qCPA secure with a classical adaptive nonce N (N cannot be repeated).*

**Proof.**  We prove this proposition using the game-playing technique. (Table 2).

$G_{0}$: The adversary is given oracle access to the quantum oracle of $Enc_{K_{1},K_{2}}^{\prime }\left(N,P\right)=Enc_{K_{2}}\left(E_{K_{1}}\left(N\right),P\right)$.

$G_{1}$: We change *E* to ideal random function *f*. Let $A_{1}$ be adversary run to the quantum oracle of $G_{0}$ or $G_{1}$. Let $B_{1}$ be an adversary run to the classical oracle of *E* or random function *f*. Adversary $B_{1}$ starts by running $A_{1}$ and simulating the games $G_{0}$ and $G_{1}$ for it. In order to simulate the calls to $S_{1}$, $B_{1}$ uses its own oracles from the PRF game. Note that *N* is a classic number, and choosing a non-repeating *N* is easy. Then, adversary $B_{1}$ keeps track of all the sets appearing in the games $G_{0}$ or $G_{1}$ and enforces the corresponding game rules. In the end, adversary $B_{1}$ returns the same bit that $A_{1}$ returns. Let $A_{1}$ make at most *q* quantum queries, then let $B_{1}$ make at most *q* quantum queries. It holds that

$$\begin{array}{c} {\mathrm{Adv}}_{G_{0},G_{1}}^{\mathrm{qdist}}\left(A_{1}\right)\leq {\mathrm{Adv}}_{E}^{PRF}\left(B_{1}\right). \end{array}$$

$G_{2}$: The adversary is given oracle access to the quantum oracle of $Enc_{K_{2}}\left(IV,P\right)$. Let $A_{2}$ be an adversary run to the quantum oracle of $G_{1}$ or $G_{2}$. Let $B_{2}$ be an adversary run to the classical oracle of random function *f* or random IV. $B_{2}$ uses its own oracles to simulate $S_{1}$. Then, adversary $B_{1}$ starts by running $A_{1}$ and simulating the games $G_{1}$ and $G_{2}$ for it. Adversary $B_{1}$ keeps track of all the sets appearing in games $G_{1}$ or $G_{2}$ and enforces the corresponding game rules. In the end, adversary $B_{2}$ returns the same bit that $A_{2}$ returns. Let $A_{2}$ make at most *q* quantum queries, then let $B_{2}$ make at most *q* quantum queries. It holds that

$$\begin{array}{c} {\mathrm{Adv}}_{G_{1},G_{2}}^{\mathrm{qdist}}\left(A_{2}\right)=0. \end{array}$$

Thus, we have

$$\begin{array}{c} {\mathrm{Adv}}_{G_{0},G_{2}}^{\mathrm{qdist}}\left(A\right)\leq {\mathrm{Adv}}_{G_{0},G_{1}}^{\mathrm{qdist}}\left(A_{1}\right)+{\mathrm{Adv}}_{G_{1},G_{2}}^{\mathrm{qdist}}\left(A_{2}\right)\leq {\mathrm{Adv}}_{E}^{PRF}\left(B_{1}\right). \end{array}$$

□

## 5. N-IND-qCPA Secure Modification Modes

According to Theorem 4, CFB and OFB can be enhanced to N-IND-qCPA secure versions, which we denote as CFB2 and OFB2, respectively. Additionally, we define the improved CTR version, denoted as CTR2, which is N-IND-qCPA secure.

### 5.1. CFB2 Mode

**Figure 8:**
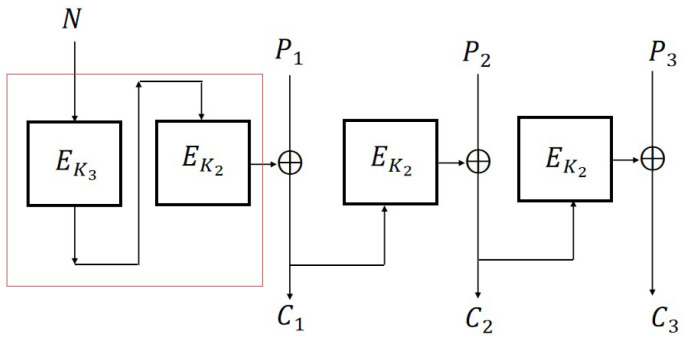
CFB2 scheme (standard construction with m=3).

We use different keys $K_{3}$ and $K_{2}$ to encrypt adaptive nonce *N* and message *P*, respectively. The improved version of CFB with N-IND-qCPA secure is shown in Figure 8.

**Figure 9:**
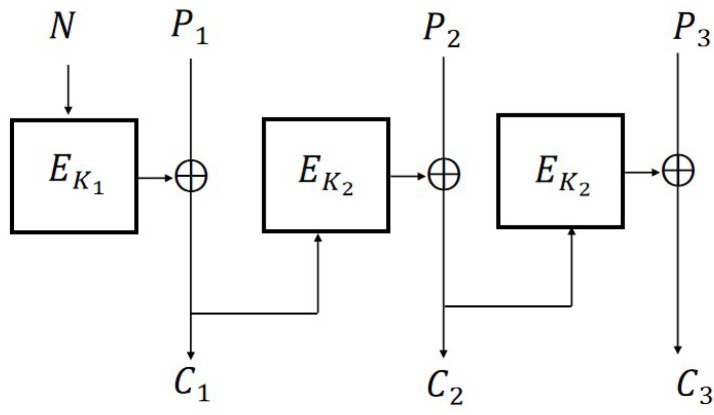
CFB2 scheme (simplified construction with m=3).

Let $E_{K_{1}}\overset{\;\mathrm{def}\;}{=}E_{K_{2}}\circ E_{K_{3}}$. We can obtain a simplified version of CFB2 (Figure 9) as follows:

**Definition 13**  (CFB2 Mode). *Let $K_{1},K_{2}\overset{\$}{\leftarrow }{\left\{0,1\right\}}^{k}$. For a given message $P=P_{1}P_{2}\ldots P_{m}$ and nonce N, where m is a polynomial in n, the symmetric encryption scheme CFB2 is defined as follows:*

*Enc: $C_{1}=E_{K_{1}}\left(N\right)⊕P_{1}$ and $C_{i}=E_{K_{2}}\left(C_{i-1}\right)⊕P_{i}$ for 2≤i≤m. En$c_{K_{1},K_{2}}\left(P\right)=C_{1}\ldots C_{m}$.*
*Dec: For a given ciphertext $C=C_{1}\ldots C_{m}$, $P_{i}:=E_{K_{2}}\left(C_{i-1}\right)⊕C_{i}$ for 2≤i≤m, $P_{1}=E_{K_{1}}\left(N\right)⊕C_{1}$. De$c_{K_{1},K_{2}}\left(C\right)=P_{1}\ldots P_{m}$.*

Theorem 4 directly implies the following theorem.

**Theorem 5.**  *If the function E is a quantum secure PRF, then CFB2 is N-IND-qCPA secure.*

### 5.2. OFB2 Mode

**Figure 10:**
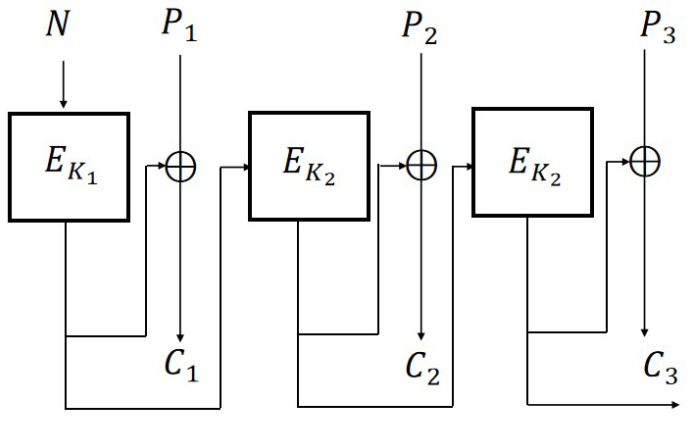
OFB2 scheme (m=3).

Similar to Section 5.1, we define the OFB2 scheme (Figure 10) as follows:

**Definition 14**  (OFB2 Mode). *Let $K_{1},K_{2}\overset{\$}{\leftarrow }{\left\{0,1\right\}}^{k}$. For a given message $P=P_{1}P_{2}\ldots P_{m}$ and nonce N, where m is a polynomial in n, the symmetric encryption scheme OFB2 is defined as follows:*

*Enc: $R_{0}=E_{K_{1}}\left(N\right)$, $R_{i}=E_{K_{2}}\left(R_{i-1}\right)$ for 2≤i≤m. Then $C_{i}=R_{i-1}⊕P_{i}$ for 1≤i≤m. En$c_{K_{1},K_{2}}\left(P\right)=C_{1}\ldots C_{m}$.*
*Dec: For a given ciphertext $C=C_{1}\ldots C_{m}$, $C_{0}=E_{K_{1}}\left(N\right)$, $P_{1}=C_{0}⊕C_{1}$, $P_{i}:=E_{K_{2}}\left(C_{i-1}\right)⊕C_{i}$ for 2≤i≤m. De$c_{K_{1},K_{2}}\left(C\right)=P_{1}\ldots P_{m}$.*

Theorem 4 directly implies the following theorem.

**Theorem 6.**  *If the function E is a quantum secure PRF, then OFB2 is N-IND-qCPA secure.*

### 5.3. CTR2 Mode

**Figure 11:**
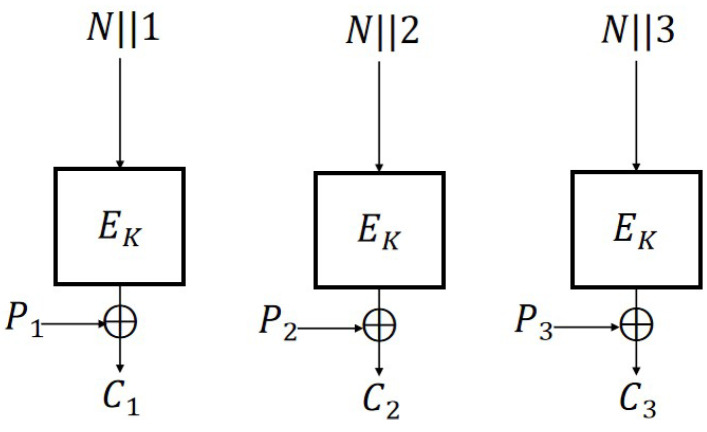
CTR2 scheme (m=3).

Because each message block of CTR2 has nonce *N* as an input, directly referencing the conclusion of Theorem 4 would lead to a significant increase in the number of keys. Therefore, we consider making improvements in terms of the input. We divide the input into two parts, *N* and i,i=1,2,3…, and concatenate them directly. Correspondingly, the length of the block cipher $E_{K}$ used at this time is 2n, and the lengths of $P_{i}$ and $C_{i}$ are also 2n. We define the N-IND-qCPA secure CTR2 scheme (Figure 11) as follows:

**Definition 15**  (CTR2 Mode). *Let $K\overset{\$}{\leftarrow }{\left\{0,1\right\}}^{k}$. For a given message $P=P_{1}P_{2}\ldots P_{m}$ and nonce N, where m is a polynomial in n, the symmetric encryption scheme CTR2 is defined as follows:*

*Enc: $C_{0}=N$ and $C_{i}=E_{K}\left(C_{0}∥i\right)⊕P_{i}$ for 1≤i≤m. En$c_{K}\left(P\right)=C_{1}\ldots C_{m}$.*
*Dec: For a given ciphertext $C=C_{1}\ldots C_{m}$ and $C_{0}=N$, $P_{i}:=E_{K}\left(C_{0}∥i\right)⊕C_{i}$ for 1≤i≤m. De$c_{K}\left(C\right)=P_{1}\ldots P_{m}$.*

For the CTR2 scheme, the following theorem holds:

**Theorem 7.**  *If the function E is a secure PRF, then CTR2 is N-IND-qCPA secure.*

**Proof.**  We prove this proposition using the game-playing technique. (Table 3).

$G_{0}$: The adversary is given oracle access to the quantum oracle of CTR2.

$G_{i},2\leq i\leq m$: We change the *i*th *E* to ideal random function *f*. Let $A_{i}$ be an adversary run to the quantum oracle of $G_{i-1}$ or $G_{i}$. Let $B_{i}$ be an adversary run to the classical oracle of *E* or random function *f*. Adversary $B_{i}$ starts by running $A_{i}$ and simulating the games $G_{i-1}$ and $G_{i}$ for it. In order to simulate the calls to $S_{1}^{i}$, $B_{i}$ uses its own oracles from PRF game. Note that *N* is a classic number, and choosing a non-repeating *N* is easy. Then, adversary $B_{i}$ keeps track of all the sets appearing in the games $G_{i-1}$ or $G_{i}$ and enforces the corresponding game rules. In the end, adversary $B_{i}$ returns the same bit that $A_{i}$ returns. Let $A_{i}$ make at most *q* quantum queries, then let $B_{i}$ make at most *q* quantum queries. It holds that

$$\begin{array}{c} {\mathrm{Adv}}_{G_{i-1},G_{i}}^{\mathrm{qdist}}\left(A_{i}\right)\leq {\mathrm{Adv}}_{E}^{PRF}\left(B_{i}\right). \end{array}$$

For quantum states *P*, game $G_{m}$ returns the XOR value between a quantum message block and a classical random number. Therefore, game $G_{m}$ returns a random quantum state. It holds that

$$\begin{array}{cc} {\mathrm{Adv}}_{G_{0},G_{m}}^{\mathrm{qdist}}\left(A_{i}\right) & \leq {\mathrm{Adv}}_{G_{0},G_{1}}^{\mathrm{qdist}}\left(A_{i}\right)+{\mathrm{Adv}}_{G_{1},G_{2}}^{\mathrm{qdist}}\left(A_{i}\right)+\ldots +{\mathrm{Adv}}_{G_{m-1},G_{m}}^{\mathrm{qdist}}\left(A_{i}\right)\\ \; & \leq \sum_{1\leq i\leq m}{\mathrm{Adv}}_{E}^{PRF}\left(B_{i}\right). \end{array}$$

□

We note that CTR2 is a stream cipher, and the conclusion and proof of Theorem 7 can be correspondingly extended to stream ciphers.

**Theorem 8.**  *[Nonce-based stream cipher encryption is N-IND-qCPA] Let G be a secure nonce-based stream cipher, $Enc_{K}\left(N,P\right)=G_{K}\left(N\right)⊕P$; then, $Enc_{K}\left(N,P\right)$ is N-IND-qCPA secure.*

**Proof.**  The proof of this theorem is similar to Theorem 7. We prove this proposition using the game-playing technique. (Table 4).

$G_{0}$: The adversary is given oracle access to the quantum oracle of $Enc_{K}\left(N,P\right)$.

$G_{1}$: We change *G* to an ideal random function *f*. Let $A_{1}$ be an adversary run to the quantum oracle of $G_{0}$ or $G_{1}$. Let $B_{1}$ be an adversary run to the classical oracle of *G* or random function *f*. Similar to the previous proof, we have

$$\begin{array}{c} {\mathrm{Adv}}_{G_{0},G_{1}}^{\mathrm{qdist}}\left(A_{1}\right)\leq {\mathrm{Adv}}_{G}^{PRF}\left(B_{1}\right). \end{array}$$

$G_{2}$: We change random function *f* to random $S_{1}\overset{\$}{\leftarrow }{\left\{0,1\right\}}^{|G_{K}\left(N\right)|}$. Let $A_{2}$ be an adversary run to the quantum oracle of $G_{1}$ or $G_{2}$, and let

$$\begin{array}{c} {\mathrm{Adv}}_{G_{1},G_{2}}^{\mathrm{qdist}}\left(A_{2}\right)=0. \end{array}$$

Thus, we have

$$\begin{array}{c} {\mathrm{Adv}}_{G_{0},G_{2}}^{\mathrm{qdist}}\left(A\right)\leq {\mathrm{Adv}}_{G_{0},G_{1}}^{\mathrm{qdist}}\left(A_{1}\right)+{\mathrm{Adv}}_{G_{1},G_{2}}^{\mathrm{qdist}}\left(A_{2}\right)\leq {\mathrm{Adv}}_{G}^{PRF}\left(B_{1}\right). \end{array}$$

For quantum states *P*, game $G_{2}$ returns the XOR value between a quantum message block and a classical random number. Therefore, game $G_{2}$ returns a random quantum state, and $Enc_{K}\left(N,P\right)$ is N-IND-qCPA secure. □

Note that if OFB2 is also a stream cipher, the N-IND-qCPA security of OFB2 (Theorem 6) can also be directly derived from Theorem 8.

## 6. Conclusions

The first contribution of this paper is a general conversion method from R-IND-qCPA to N-IND-qCPA security (Theorem 4). Specifically, if an encryption scheme $Enc_{K_{2}}\left(IV,P\right)$ is R-IND-qCPA secure with random IV, then the construction $Enc_{K_{2}}\left(E_{K_{1}}\left(N\right),P\right)$ achieves N-IND-qCPA security with adaptive but non-repeating nonce *N*. Conceptually, this transformation encrypts the adaptive nonce once, thereby turning it into a random IV. As a direct application, CBC2 is proven N-IND-qCPA secure. We note, however, that this method generally incurs one additional encryption operation.

In contrast, for CFB and OFB, the situation is more favorable; since these schemes already involve encrypting the IV once, the additional operation can be merged. Thus, the modified variants CFB2 and OFB2 achieve N-IND-qCPA security without extra computational overhead, requiring only distinct keys for the initial block and the subsequent blocks.

Our final result establishes that nonce-based stream ciphers are inherently N-IND-qCPA secure (Theorem 8). If *G* is a secure nonce-based stream cipher, then $G_{K}\left(N\right)⊕P$ satisfies N-IND-qCPA security. This can be understood as XORing quantum plaintext states with classical randomness. As a corollary, we derive the N-IND-qCPA secure version CTR2.

When compared to random IVs, nonces that are selectable yet non-repeating offer better alignment with practical deployment requirements. Future research should focus on extending these techniques to further enhance the N-IND-qCPA security of other IV-based encryption schemes. Meanwhile, integrating confidentiality with integrity to develop quantum-secure authenticated encryption schemes represents another important direction for future research.

## Figures and Tables

**Table 1:** R-IND-CPA, N-IND-CPA, R-IND-qCPA, N-IND-qCPA security of encryption schemes.

Encryption Schemes	R-IND-CPA	N-IND-CPA	R-IND-qCPA	N-IND-qCPA	Ref.
CBC	yes	no	yes	no	[12], Section 3
CFB	yes	no	yes	no	[12], Section 3
OFB	yes	no	yes	no	[12], Section 3
CTR	yes	no	yes	no	[12], Section 3
CBC2	yes	yes	yes	yes	Section 4
CFB2	yes	yes	yes	yes	Section 5
OFB2	yes	yes	yes	yes	Section 5
CTR2	yes	yes	yes	yes	Section 5

**Table 2:** The games $G_{0}$, $G_{1}$ and $G_{2}$.

Game $G_{0}$	Game $G_{1}$	Game $G_{2}$
**Initialization**	**Initialization**	**Initialization**
$K_{1},K_{2}\overset{\$}{\leftarrow }{\left\{0,1\right\}}^{k}$	$K_{1},K_{2}\overset{\$}{\leftarrow }{\left\{0,1\right\}}^{k}$	$K_{2}\overset{\$}{\leftarrow }{\left\{0,1\right\}}^{k}$
**On query** $\left(N,\sum_{P}\psi_{P}|P\rangle \right)$	**On query** $\left(N,\sum_{P}\psi_{P}|P\rangle \right)$	**On query** $\left(IV,\sum_{P}\psi_{P}|P\rangle \right)$
$S_{1}=E_{K_{1}}\left(N\right)$	$S_{1}=f_{K_{1}}\left(N\right)$	$S_{1}=IV$
$\sum_{P,S_{2}}\psi_{P,S_{2}}|P,S_{2}\rangle =$	$\sum_{P,S_{2}}\psi_{P,S_{2}}|P,S_{2}\rangle =$	$\sum_{P,S_{2}}\psi_{P,S_{2}}|P,S_{2}\rangle =$
$\sum_{P,S_{2}}\psi_{P,S_{2}}|P,S_{2}⊕Enc_{K_{2}}\left(S_{1},P\right)\rangle$	$\sum_{P,S_{2}}\psi_{P,S_{2}}|P,S_{2}⊕Enc_{K_{2}}\left(S_{1},P\right)\rangle$	$\sum_{P,S_{2}}\psi_{P,S_{2}}|P,S_{2}⊕Enc_{K_{2}}\left(S_{1},P\right)\rangle$
return $\sum_{S_{2}}\psi_{S_{2}}|S_{2}\rangle$	return $\sum_{S_{2}}\psi_{S_{2}}|S_{2}\rangle$	return $\sum_{S_{2}}\psi_{S_{2}}|S_{2}\rangle$

**Table 3:** The games $G_{0}$, $G_{1}$,…,$G_{m}$.

Game $G_{0}$	Game $G_{1}$	…	Game $G_{m}$
**Initialization**	**Initialization**	…	**Initialization**
$K\overset{\$}{\leftarrow }{\left\{0,1\right\}}^{k}$	$K\overset{\$}{\leftarrow }{\left\{0,1\right\}}^{k}$	…	$K\overset{\$}{\leftarrow }{\left\{0,1\right\}}^{k}$
**On query** $\left(N,\sum_{P}\psi_{P}|P\rangle \right)$	**On query** $\left(N,\sum_{P}\psi_{P}|P\rangle \right)$	…	**On query** $\left(N,\sum_{P}\psi_{P}|P\rangle \right)$
	$S_{1}^{1}=f_{K}\left(N∥1\right)$	…	
	$\sum_{P_{1},S_{2}^{1}}\psi_{P_{1},S_{2}^{1}}|P_{1},S_{2}^{1}\rangle =$		
	$\sum_{P_{1},S_{2}^{1}}\psi_{P_{1},S_{2}^{1}}|P_{1},S_{2}^{1}⊕S_{1}^{1}⊕P_{1}\rangle$		
for 1≤i≤m	for 2≤i≤m	…	for 1≤i≤m
$S_{1}^{i}=E_{K}\left(N∥i\right)$	$S_{1}^{i}=E_{K}\left(N∥i\right)$	…	$S_{1}^{i}=f_{K}\left(N∥i\right)$
$\sum_{P_{i},S_{2}^{i}}\psi_{P_{i},S_{2}^{i}}|P_{i},S_{2}^{i}\rangle =$	$\sum_{P_{i},S_{2}^{i}}\psi_{P_{i},S_{2}^{i}}|P_{i},S_{2}^{i}\rangle =$		$\sum_{P_{i},S_{2}^{i}}\psi_{P_{i},S_{2}^{i}}|P_{i},S_{2}^{i}\rangle =$
$\sum_{P_{i},S_{2}^{i}}\psi_{P_{i},S_{2}^{i}}|P_{i},S_{2}^{i}⊕S_{1}^{i}⊕P_{i}\rangle$	$\sum_{P_{i},S_{2}^{i}}\psi_{P_{i},S_{2}^{i}}|P_{i},S_{2}^{i}⊕S_{1}^{i}⊕P_{i}\rangle$		$\sum_{P_{i},S_{2}}\psi_{P_{i},S_{2}}|P_{i},S_{2}⊕S_{1}^{i}⊕P_{i}\rangle$
end for	end for	…	end for
return $\sum_{S_{2}^{1},\ldots ,S_{2}^{m}}\psi_{S_{2}^{1},\ldots ,S_{2}^{m}}|S_{2}^{1},\ldots ,S_{2}^{m}\rangle$	return $\sum_{S_{2}^{1},\ldots ,S_{2}^{m}}\psi_{S_{2}^{1},\ldots ,S_{2}^{m}}|S_{2}^{1},\ldots ,S_{2}^{m}\rangle$	…	return $\sum_{S_{2}^{1},\ldots ,S_{2}^{m}}\psi_{S_{2}^{1},\ldots ,S_{2}^{m}}|S_{2}^{1},\ldots ,S_{2}^{m}\rangle$

**Table 4:** The games $G_{0}$, $G_{1}$ and $G_{2}$.

Game $G_{0}$	Game $G_{1}$	Game $G_{2}$
**Initialization**	**Initialization**	**Initialization**
$K\overset{\$}{\leftarrow }{\left\{0,1\right\}}^{k}$	$K\overset{\$}{\leftarrow }{\left\{0,1\right\}}^{k}$	$K\overset{\$}{\leftarrow }{\left\{0,1\right\}}^{k}$
**On query** $\left(N,\sum_{P}\psi_{P}|P\rangle \right)$	**On query** $\left(N,\sum_{P}\psi_{P}|P\rangle \right)$	**On query** $\left(N,\sum_{P}\psi_{P}|P\rangle \right)$
$S_{1}=G_{K}\left(N\right)$	$S_{1}=f_{K}\left(N\right)$	$S_{1}\overset{\$}{\leftarrow }{\left\{0,1\right\}}^{|G_{K}\left(N\right)|}$
$\sum_{P,S_{2}}\psi_{P,S_{2}}|P,S_{2}\rangle =$	$\sum_{P,S_{2}}\psi_{P,S_{2}}|P,S_{2}\rangle =$	$\sum_{P,S_{2}}\psi_{P,S_{2}}|P,S_{2}\rangle =$
$\sum_{P,S_{2}}\psi_{P,S_{2}}|P,S_{2}⊕S_{1}⊕P\rangle$	$\sum_{P,S_{2}}\psi_{P,S_{2}}|P,S_{2}⊕S_{1}⊕P\rangle$	$\sum_{P,S_{2}}\psi_{P,S_{2}}|P,S_{2}⊕S_{1}⊕P\rangle$
return $\sum_{S_{2}}\psi_{S_{2}}|S_{2}\rangle$	return $\sum_{S_{2}}\psi_{S_{2}}|S_{2}\rangle$	return $\sum_{S_{2}}\psi_{S_{2}}|S_{2}\rangle$

## Data Availability

No new data were created or analyzed in this study. Data sharing is not applicable to this article.

## Abbreviations

The following abbreviations are used in this manuscript:

IND-CPA	Indistinguishability under Chosen-Plaintext Attack
IND-qCPA	Indistinguishability under quantum Chosen-Plaintext Attack
IV	Initialization Vector
N	Nonce
R-IND-CPA	Indistinguishability under Chosen-Plaintext Attack in the random-IV setting
N-IND-CPA	Indistinguishability under Chosen-Plaintext Attack in the nonce setting
R-IND-qCPA	Indistinguishability under quantum Chosen-Plaintext Attack in the random-IV setting
N-IND-qCPA	Indistinguishability under quantum Chosen-Plaintext Attack in the nonce setting
CBC	Cipher Block Chaining
CFB	Cipher FeedBack Mode
OFB	Output FeedBack Mode
CTR	CounTeR Mode
